# An integrated, multidisciplinary management team intervention to improve patient-centeredness, HIV, and maternal-child outcomes in Lesotho: formative research on participatory implementation strategies

**DOI:** 10.1186/s12913-024-12049-x

**Published:** 2024-12-18

**Authors:** Laura K. Beres, Mammatli Chabela, Matseliso Masitha, Zachary Catanzarite, Vincent J. Tukei, Lynne Mofenson, Appolinaire Tiam, Lauren Greenberg, Majoalane Mokone, Ramatlapeng Thabelo, Masepeli Nchephe, Tsietso Mots’oane, Laura Guay, Amy R. Knowlton

**Affiliations:** 1 Johns Hopkins University, Bloomberg School of Public Health, Department of International Health, Baltimore, MD USA; 2 Elizabeth Glaser Pediatric AIDS Foundation, Maseru, Lesotho; 3 Johns Hopkins University, Bloomberg School of Public Health, Department of Health, Behavior and Society, Baltimore, MD USA; 4 Elizabeth Glaser Pediatric AIDS Foundation, Washington, D.C USA; 5 Ministry of Health, Maseru, Lesotho; 6 George Washington University, Milken Institute School of Public Health, Department of Epidemiology and Biostatistics, Washington, D.C USA

**Keywords:** HIV/AIDS, Multidisciplinary care team, Care coordination, Maternal and child health, Mother-to-child HIV transmission, Antiretroviral therapy (ART) adherence, Retention in care, Patient-centered antenatal and postnatal care

## Abstract

**Background:**

Reducing perinatal HIV transmission and optimizing maternal and child health (MCH) outcomes in high HIV prevalence settings is an urgent, but complex, priority. Extant interventions over-emphasize individual-level provider and patient behaviors, and neglect critical health systems-level changes. The ‘Integrated Management Team to Improve Maternal-Child Outcomes (IMPROVE)’ study implemented a three-part, patient-centered, health-systems-level intervention to improve MCH and HIV outcomes in Lesotho. Ensuring intervention fit within the health systems context is important, but often overlooked. This manuscript describes implementation research conducted to tailor and adapt intervention implementation to optimize appropriateness, acceptability, and feasibility. It identifies resulting implementation variation across study sites and lessons learned.

**Methods:**

The research team reviewed intervention implementation documentation and conducted structured reflections to: 1) assess implementation strategy adaptations, 2) identify facility-specific strategies employed to improve the MCH patient experience, and 3) synthesize lessons.

**Results:**

Facility-based, integrated, multi-disciplinary management teams (MDT) were feasible and acceptable to establish through engagement with facility leadership and facilitation of a participatory training curriculum that established shared values between cadres supporting MCH, and identified facility-specific service delivery gaps and potential solutions. Ongoing MDT meetings provided coordination between facility and community-based MCH service providers to implement early ANC follow-up. Facility-specific improvement strategies included fee, staffing, and patient documentation-based changes. Piloting Positive Health, Dignity, and Prevention-focused counseling approaches resulted in tailored job aids pre-implementation. Leadership involvement was critical for improved coordination while staff turnover and competing donor priorities challenged MDT efforts.

**Conclusions:**

IMPROVE created facility-specific adaptation opportunities through participatory intervention implementation practices. The MDTs, benefitting from leadership support, built relationships between HCW cadres, led facility-specific quality improvements, and, importantly, offered HCWs sought-after positive feedback by recognizing HCW efforts. The coordination, monitoring and cross-cadre communication functions of the MDTs supported implementation of other interventions, and may serve as a valuable platform for improving patient-centered care practices in similar settings and for other health services. Trial registration number: NCT04598958, 05 October 2020, retrospectively registered.

**Trial registration:**

ClinicalTrials.gov, NCT04598958. Registered 05 October 2020—Retrospectively registered, https://clinicaltrials.gov/ct2/show/record/NCT04598958

## Contributions to the literature

- How interventions are implemented is critical to if they are successful. Often, implementation strategies focus on individual-level changes that must be enacted by health providers and patients. This individual-level focus neglects the critical role health systems and contexts play in the successful implementation of evidence-based interventions.
- Tailoring interventions and implementation strategies is increasingly recognized as important for intervention fit. However, the practice of tailoring is not universal and, when done, is often time and resource-intensive for participants. This makes understanding tailoring practices and outcomes important for public health.
- Our study demonstrates an integrated approach to tailoring implementation of a maternal and child health focused health-systems-level intervention through: 1) participatory implementation, and 2) stakeholder identification of health facility-specific priorities.
- Lessons on feasibility, acceptability, and outcome variation from this study can inform similar tailoring approaches and support increased attentiveness to health systems-level action targets in implementation science.

## Background

Optimizing maternal and child health (MCH) outcomes in high HIV prevalence settings is an urgent, but complex, priority. While an estimated 95% of women living with HIV accessed antiretroviral therapy (ART) during pregnancy in eastern and southern Africa [1], approximately one-quarter to one-half of pregnant and postpartum African women living with HIV are not retained in HIV care [2]. Countries including Lesotho have made substantial gains in reducing mother-to-child (MTCT) HIV transmission from approximately 8% to 5% since 2018; however, further reductions are needed to achieve established MTCT elimination goals [3, 4]. Infant HIV diagnosis requires testing at several time points during the first 18 months of life and remains weak [1]. Improving MCH outcomes requires: 1) widespread HIV prevention in HIV-negative, at-risk pregnant and breastfeeding women, 2) early, sustained ART among pregnant and breastfeeding women living with HIV, and 3) broad utilization of evidence-based MCH services such as facility-based delivery and child immunization to improve well-being. Barriers to realizing the full potential of efficacious MCH and HIV tools span individual, social, structural, and health systems-level challenges including limited care accessibility and quality [5, 6]. There is evidence that integrated, patient-centered care improves patient engagement and a range of health outcomes [7–11], though few studies have examined its effects on MCH outcomes in high prevalence HIV settings. Implementation generally overemphasizes individual agency in making change, neglecting higher-level influences critical to fostering action [12]. Multi-level interventions tailored and acceptable to both patients and health care worker implementers that are also appropriate for their health systems and community context are urgently needed.

The ‘Integrated Management Team to Improve Maternal-Child Outcomes (IMPROVE)’ Study evaluated a patient-centered, health systems-level intervention to increase MCH, ART, and HIV services uptake and retention for improved MCH outcomes in Lesotho, southern Africa. The intervention included three evidence-based, integrated interventions: 1) multi-disciplinary management teams to improve coordination of MCH and prevention of mother-to-child HIV transmission (PMTCT) services at facilities [13, 14], 2) enhanced Positive Health, Dignity, and Prevention (PHDP)-focused counseling to better center people, not HIV, within HIV prevention and treatment as guided by the framework established by persons living with HIV [15, 16], 3) increased early, community-based counseling and support for antenatal care (ANC) attendees (including 1–2 additional home or community visits at 2–7 days and/or 9–14 days after the first ANC visit) [17]. Extant literature supports tailoring implementation strategies to fit contextual realities to achieve optimal health impacts [18, 19]. This may be particularly valuable for implementation of integrated, health systems-level interventions [20–22] and patient-centered approaches [23]; however, there is limited research articulating the tailoring processes or results.

The IMPROVE study conducted formative research utilizing a facility-specific, participatory approach to tailor and adapt implementation of the integrated, patient-centered, health systems intervention to improve PMTCT and MCH outcomes. The implementation evaluation sought to assess intervention appropriateness, acceptability, and feasibility, alongside implementation variability across study sites. This manuscript describes adaptation of the implementation of evidence-based intervention components and implementation results.

## Methods

### Study context

The formative research component tailored the implementation of the integrated intervention to improve patient-centered care tested in the IMPROVE study. IMPROVE was a cluster-randomized trial comparing outcomes between six intervention and six standard-of-care (control) facilities in Maseru District, Lesotho. Outcomes were assessed prospectively, following an enrolled cohort of pregnant women attending their first ANC visit through 24 months postpartum. For women living with HIV the following primary study outcomes were investigated: retention in care, ART adherence, and viral suppression. For women not living with HIV the primary study outcome was HIV retesting. For women and infants, primary study outcomes investigated were: proportion with health facility-based delivery, and complete infant immunizations by 24 months of age. A range of secondary endpoints were also evaluated, including MCH, HIV, depressive symptoms, cost, and implementation outcomes. Health facilities eligibility criteria were: 1) located in Maseru District; 2) provided PMTCT, early infant HIV diagnosis (EID), and MCH services; 3) annual volume of 150 – 900 ANC attendees, and 4) managed by the Government of Lesotho or Christian Health Association of Lesotho (CHAL).

The study was implemented from July 2016 – July 2019 by investigators from the Lesotho Ministry of Health (MOH), the Elizabeth Glaser Pediatric AIDS Foundation (EGPAF), the National University of Lesotho, and Project SOAR (Supporting Operational AIDS Research) partners affiliated with Johns Hopkins and George Washington Universities and Avenir Health. Serving as a longstanding, collaborative implementing partner, EGPAF has supported the MOH in adult and pediatric HIV service provision throughout Lesotho since 2004.

### Study setting

Lesotho is a mountainous country landlocked by South Africa, population just over 2 million [24]. Lesotho has the second highest adult (15–49 years) HIV prevalence in the world at 24.3% [25] and among the highest maternal and child mortality rates [26]. While uptake of ART among HIV positive pregnant women is greater than 90%, approximately 20% are not virally suppressed [25]. There were an estimated 790 new pediatric HIV infections in 2019 [1], and an estimated vertical transmission rate of 5.01 (3.36-6.85) including during breastfeeding [27]. Maseru, the study district which includes the national capital, has the second highest HIV prevalence rate in the country (27.8%; 29.5% in pregnant women) and the second lowest rate of viral suppression (64.8%) [25]. Overall, health facilities in Maseru, spanning both urban and mountainous hard-to-reach areas have especially high antenatal patient volume, and low coverage of prevention of PMTCT and MCH services.

Lesotho’s healthcare system provides free ANC and HIV testing and treatment at health facilities and in the community. At the time of the study Lesotho MOH’s recommended standard of care for pregnant and postpartum women included a minimum of 4 antenatal visits, facility delivery, and maternal visits at 7-days, 6-weeks, and 14-weeks postpartum. Additional visits were recommended for family planning (as desired), HIV re-testing among women testing negative (10–14 weeks after initial test and then yearly retesting is recommended), ART follow-up (2 weeks after starting ART, with subsequent visits at 1, 2, 3, 6 months and then every 3 months until the child is age 24 months), and early infant HIV testing through 18 months among women living with HIV. Ministry of Health-supported Village health workers (VHWs) and non-governmental organizations (NGOs) including Mothers2Mothers (M2M) and the Lesotho Network of AIDS Service Organizations (LENASO) provide targeted, community-based health support emphasizing different types of care (e.g., ANC, MCH, HIV), with varying degrees of coordination through the MOH and local health facility structures. The community partners had different but overlapping focus areas: VHWs emphasized ANC and MCH without a focus on HIV-related services; M2M, an organization engaging local community-based women living with HIV as peer community health workers [28], also focused on ANC and MCH but with an emphasis on PMTCT and maternal ART; LENASO primarily focused on maternal ART, but not PMTCT. This variation in coordination led to inconsistent coverage of the full range of relevant HIV, ANC, and MCH support services at the community level.

### Intervention conceptual framework and implementation approach

**Fig. 1:**
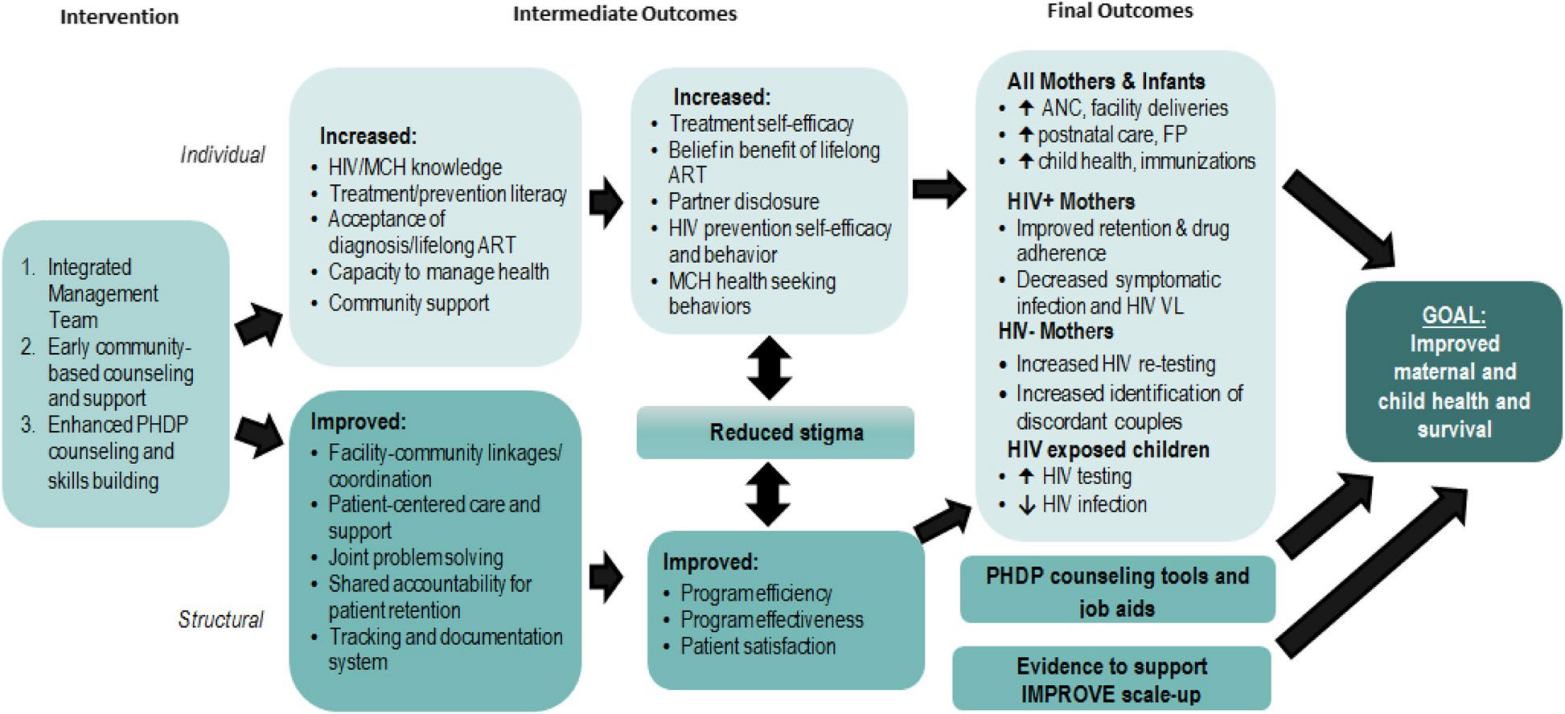
Study conceptual framework

The study intervention goal was to increase overall MCH, ART, and HIV service uptake and retention. The three-part intervention (integrated multi-disciplinary management teams; enhanced PHDP counseling; and early community-based counseling and support) was designed to improve person-centeredness of HIV care through influencing individual (e.g. increased knowledge, capacity, support) and structural / health system behavioral targets (e.g. improved care coordination, partnership with patients) [29] to achieve the desired intermediate and final outcomes (Fig. 1) through engaging key components of successful interdisciplinary care [14, 30]. It addressed services delivered through general MCH, including ANC and post-natal care (PNC); family planning (FP); PMTCT; and pediatric ART services. To promote feasibility of intervention scale-up if successful, aside from funds for initial training workshops, the study built on existing health system infrastructure and resources and did not provide additional staff members (professional or lay health workers) or additional finances for ongoing implementation of intervention components.

IMPROVE intervention implementation leveraged existing collaborations between EGPAF, MOH, CHAL, LENASO, and Lesotho District Health Management Team. Study-specific stakeholder engagement occurred early during protocol planning and continued through implementation and result dissemination. This included outreach from EGPAF to M2M to discuss M2M priorities, identify alignment with study goals, and agree to a partnership. The study was vetted by collaborating stakeholders (i.e., the MOH PMTCT Technical Working Group and MOH AIDS Development Partners Forum, including representatives from UN agencies, the US Centers for Disease Control and Prevention, and USAID), to elicit specific feedback and to gain stakeholder support.

The study team theorized tailoring intervention implementation based on formative research and facilitating facility-led adaptation of the planned implementation would improve its appropriateness, acceptability, and feasibility.

### Adaptation of intervention content and implementation strategies

The intervention was tailored to the Lesotho context through formative research with 1) the six facility-level multidisciplinary management teams of intervention implementers linked to the six study intervention sites (healthcare facilities), 2) MOH and other government officials, and 3) nurse-led EGPAF-associated research team members. Facility-level multidisciplinary teams, called Multidisciplinary Care Management Teams (MDTs), included health facility leadership, professional healthcare workers in MCH, PMTCT, pharmacy, health facility administrative staff, lay counselors, VHWs, and workers from M2M and LENASO serving in the intervention facility catchment.

The interlinked intervention components were implemented through targeted strategies, each allowing for facility-level adaptation to improve appropriateness, acceptability, and feasibility based on facility-level variation in MCH challenges and opportunities. Central to the implementation strategies was engagement of each facility’s service providers via the integrated, Multidisciplinary Care Management Teams (MDTs). Each MDT, comprised of facility- and community-based MCH service delivery representatives, adapted and implemented their site-specific package of interventions through a facilitated, participatory process. This began in a training workshop, described below, and was reinforced through post-training, ongoing MDT mentoring. The MDT guided implementation of the other intervention components throughout the study. The goal of all MDTs was to establish regular communication and coordination among facility MCH and PMTCT service providers, and between facility-based and community-based service providers (e.g., VHWs, LENASO, M2M) to provide more patient-centered care. MDTs were also tasked with leading implementation of solutions to the facility-specific priorities identified in the training workshops intended to improve patient-centeredness.

#### Workshop-delivered training curriculum: establishment of MDT, Enhanced PHDP counseling, facility-community coordination for early community-based counseling and support

To establish the MDT, promote patient-centeredness, PHDP skills, and improve coordination in MCH/PMTCT care, a highly interactive one-and-a-half-day training workshop was conducted at each study site. Trainings were co-led by the lead nurse interventionist and a senior facility administrator then delivered to care teams comprising all MCH-associated professional and lay healthcare workers (HCW). Objectives were to: 1) sensitize staff to high risk pregnant women’s healthcare experiences navigating care and associated care outcomes, 2) establish shared values amongst care providers around patient-centered care and commitment to working collaboratively to improve women’s experiences and outcomes, 3) adopt a systems-level perspective of factors that impede or facilitate MCH care access and retention, and 4) pilot key messages to support improved patient-provider communication.

Workshop curriculum covered patient-centered values and counseling skills, as well as team problem solving skills and was presented using participatory exercises to enhance engagement (e.g., role plays, modeling, humor, positive and negative framing). Group reflection on lived experiences of team members and clinic patients was used to achieve collective recognition of the value of each patient within their circumstances and a commitment to a common mission to achieve patient-centered care. Small (multidisciplinary) group activities were used to: 1) map patient flow through care points within the facility and the community, 2) identify problematic points of care and potential ways to improve patient experiences (flow, quality, and retention) in care; 3) adopt and practice job aids for tailored, empathic counseling; and 4) identify HCWs’ disparate documentation and communication tools and ways to potentially harmonize their use in the field.

In the workshop, each MDT identified 2–3 readily implementable strategies to improve patient experiences. MDTs also committed to meet again post-workshop to coordinate, implement, and evaluate strategies. Structured exercises elicited each team members’ articulation of their role, formal and informal, in MCH and HIV activities. Group discussions specified task shifting roles to better implement strategies for improving women’s care experiences within the facility, and better integrate delivery of MCH and HIV services in community settings.

Prior to launch of the workshops, a team comprising a PhD-trained social and behavioral scientist, lead nurse interventionist, EGPAF investigators, and MOH curricula designers conducted a 3-day training-of-trainers (TOT) in June 2016. The TOT team developed the first drafts of the curricula and other study materials. During the TOT, intervention materials were refined by the HCWs being trained as trainers. To refine the materials, the main facilitator encouraged the HCW participants to apply real life situations and encountered experiences to frame the concepts, with the goal of improving curriculum resonance and appropriateness to the facility and community contexts. Finalized materials were then used by the EGPAF nurse interventionist to serially co-facilitate the training with a facility-based HCW at each of the intervention facilities. The interactive training approach encouraged HCWs to apply the core curriculum concepts through exploration of their own, facility-specific experiences. With particular attention to patient groups having poor retention and outcomes, the training facilitated HCWs across cadres to collectively identify strategies to improve service delivery.

#### Job aids: enhanced PHDP counseling

To support consistency of counselling messages across facility and community-based service providers, PHDP job aids with PMTCT ‘key messages’ were introduced and practiced at intervention sites as part of the training. The job aids comprised linked pocket-sized cards of individualized counseling scripts. Scripts were tailored to women who were HIV-negative; newly diagnosed with HIV; initiating ART; already on ART; or in an HIV-discordant relationship. The brief messages were intended to improve women’s engagement and retention in care, self-efficacy to adhere to ART, support for partner HIV status disclosure, and connection to other clinical and community-based services [8]. All intervention MCH and PMTCT facility and community-based providers were trained to use job aids through both didactic and interactive role play and reflection modalities.

The IMPROVE study team and the PMTCT and Health Education units of the MOH worked collaboratively to develop and refine the job aids. The key messages were then piloted with HCWs at the TOT, described above, and with patients by TOT-trained HCWs. Feedback during the pilot phases was used to tailor the messages to improve appropriateness and acceptability.

#### MDT Support and coordination: establishment of MDT, facility-community coordination for early community-based counseling and support

Implementation strategies to support establishment of the MDTs and MDT operations included early engagement of facility leadership and mentorship from the IMPROVE Study Intervention Coordinator. To lay the foundation for the MDTs, IMPROVE study team representatives met with leadership at each intervention arm facility, supported by letters of study approval from the MOH, Maseru District Health Management Team, and CHAL. They introduced the study, the MDT concept, and asked them to invite all their MCH/PMTCT staff to the training including: MCH nurses, midwives, counsellors, pharmacy staff, laboratory staff, administrators, VHWs, LENASO and M2M. They also requested that post-training, the leadership at the facility, including Nursing Services Manager, Primary Health Care Coordinator and MCH nurses, organize a meeting of all health facility staff to: 1) Describe the IMPROVE study objectives and components, 2) Determine facility-specific MDT membership (total number and which cadres would be represented) and define membership roles and responsibilities, 3) Review existing gaps in the care continuum across MCH service points within the facility and between the facility- and community-based settings identified by the MDT in the workshop, 4) Identify the documentation systems used or needed for HCWs at each service point within the health facility and community settings, and 5) Determine viable ways of harmonizing documentation systems and coordinate service delivery at each point in the continuum of patient care and follow-up. Subsequent MDT meetings further defined the operational roles of MDT members, created facility-specific Standard Operating Procedures (SOPs) to guide MDT functioning, and developed and operationalized facility-specific activities to improve the patient experience.

The IMPROVE Study Intervention Coordinator conducted regular mentoring and facilitation meetings to support collaborative leadership and facility-level change through the MDT. The Study Coordinator observed MDT meetings, initially attending every meeting, then tapering attendance to end approximately six months prior to the study’s conclusion, while continuing to review MDT meeting minutes to understand progress and challenges. During facility visits, the Study Coordinator offered verbal support for facility efforts, asked questions to clarify and re-direct MDT plans, and shared relevant experiences from MDTs at other facilities to promote knowledge exchange. As attendance tapered, the Study Coordinator also offered suggestions for integrating patient-centered care and care coordination principles into other routine facility meetings, such as convening monthly VHW-facility meetings.

Once the MDTs were established, they were, themselves, an implementation strategy to ensure community-based follow-up of all ANC attendees in the days immediately after the first ANC visit. To implement this, MDTs were charged with coordinating consultative meetings between each facility Primary Health Care unit, VHWs, LENASO and M2M to determine who would be the appropriate cadre(s) to conduct the additional home visits, harmonize the systems for documenting patient visits and follow-up in the facility catchment, and monitor outcomes.

### Measurements and analysis

Our evaluation utilized two data sources that documented the process of intervention implementation: 1) The IMPROVE Study Intervention Coordinator’s mentorship log which included detailed notes recorded by the Study Coordinator after each facility visit or study event. This log reflected all trainings and approximately weekly visits to each trial site for the first six-months of the intervention and approximately monthly visits thereafter through the end of the study, and 2) formal MDT monthly meeting minutes from each facility. Led by the study Principal Investigators and the IMPROVE Study Intervention Coordinator, the research team met approximately six times between mid-2018 and late-2019 for structured review of the available data to: 1) assess intervention adaptations made to improve appropriateness, acceptability and feasibility of intervention components, 2) identify facility-specific strategies employed to improve the MCH/PMTCT patient experience, and 3) synthesize implementation lessons learned. Sessions were dialogue based, guided by the named topics, and grounded in documented information from the named data sources. When points of interest were not clear during a meeting the Study Coordinator would review data sources between meetings and share information over email for further discussion. Conclusions were reached based on dialogue and consensus.

### Reflexivity

The analysis team included study staff members with intimate and sustained study intervention implementation knowledge, investigators, and academic partners. While rich, the perspectives did not include community or study participant perspectives. We were guided by Standards for Reporting Implementation Science (StaRI) [31] in our write-up.

The study protocol was approved by the Lesotho National Health Research Ethics Committee, the George Washington University Institutional Review Board (IRB), and the Population Council IRB.

## Results

### Training curriculum and job aids

#### Feasibility

The interactive curriculum was successfully facilitated at all six intervention sites. This included training 134 HCWs (15—25 per site). To ensure administrative and management buy-in, each training was officially opened by the facility leadership or a District Health Management Team member. At the end of each training, each facility team had an action plan with identified gaps and associated strategies to improve the experiences of women and their babies when accessing MCH/PMTCT services. The training served as a shared experience allowing facility and community-based HCWs to interact and build rapport. This became a foundation for other intervention components, including MDT operations and improved facility-community coordination of maternal and child follow-up.

#### Adaptations to improve appropriateness and acceptability

The interactive nature of the curriculum allowed for facility-specific adaptation at each training. The facility-to-community patient flow maps drawn by MDTs in their training allowed for facility-specific discussion and clarification of existing protocols, procedures, and documentation practices affecting the patient and HCW experiences of MCH/PMTCT services.

Piloting of the key messages included in the job aids through patients and HCWs resulted in important changes. For example, the M2M participants updated the language referring to women living with HIV to be more positive and inclusive while HCWs and patients made messages more specific.

#### Lessons learned: enablers and challenges

The interactive, facility-specific training approach which allowed HCWs to share their own experiences and circumstances was well-received. This approach supported enthusiasm and active participation during the training and allowed for shared responsibility between HCW cadres in identification of gaps needing to be addressed. Over time, however, the application of PHDP principles and MDT operations waned. In response, the MDTs organized training for new members elected to redress staff turnover. Additionally, according to MDT monitoring, job aid use decreased over time. MDTs held one-day refresher trainings to promote job aid use through: 1) reinforcing HCW communication, 2) reviewing the role and value of patient-centered PHDP activities for patient engagement, and 3) group reflection on progress and sharing lessons learned. Formative study meetings with donors and other stakeholders facilitated improved communication and integration of service delivery across HCW cadre. Despite this, competing donor priorities challenged smooth coordination and planned MDT operations. This manifested as competing demands for time and attention to issues separate from MDT plans, and the loss of some community-based MDT members who were funded by external donors and subsequently released due to budget cuts. For example, the time of several supportive cadres was re-directed to focus exclusively on women in ANC with unsuppressed viral load by one partner and to a different geography within Lesotho by another. It was observed across facilities that support from facility leadership was an important factor in continuity of MDT-led initiatives.

### MDT operations and coordination

#### Feasibility

MDT members were selected by their department heads. The MDT chairperson coordinated departments within the facility, ensuring the selection and, when necessary due to turnover, replacement of MDT members. MDTs ranged from 15–25 members. Each MDT included representation from lay counselors, VHWs, LENASO and, except for one facility, M2M, with composition of professional HCWs varied by facility (Table 1). According to the SOPS, all MDTs met monthly.

**Table 1:** MDT Composition by Facility Type

Facility Type	Number of MDT Members	Professional Cadres Represented	Lay Cadres Represented
Hospital	17	Nurses Pharmacy Technicians Laboratory technician	LENASO M2M Lay counselors Village Health Workers
Health Center A	16	Nurses Pharmacy Technician	LENASO M2M Lay counselors Village Health Workers
Health Center B	25	Nurses Pharmacy Technician Record assistants Professional counselors	LENASO M2M Lay counselors Village Health Workers
Health Center C	20	Nurses Pharmacy Technician	LENASO M2M Lay counselors Village Health Workers
Health Center D	21	Nurses Pharmacy Technicians	LENASO M2M Lay counselors Village Health Workers
Health Center E	15	Nurses Professional Counselors	LENASO Lay counselors Village Health Workers

In all facilities, initial meetings successfully identified gaps in both communication and patient documentation practices within the facility and between facility and community-based providers, as well as initial solutions. Subsequent meetings further specified the facility’s efforts to resolve gaps and to monitor progress of solution implementation. To facilitate development of intermediate improvements, EGPAF study team periodically shared IMPROVE study outcomes, including ANC attendance, retention, viral suppression, and child immunization for the facility. Meeting minutes documented progress. Community-based follow-up and counseling following a woman’s first facility-based ANC visit was implemented by the VHWs, LENASO, and M2M staff associated with each facility. Tracking tools were harmonized so that one, MOH-endorsed tracking tool was adopted by all three of the community-based providers.

#### Adaptations to improve appropriateness and acceptability

Inherent to the intervention design, each facility developed unique membership, SOPs, and meeting schedules. They also identified health systems gaps and solutions to fit their facility. A range of challenges and solutions were identified. One MDT recognized that effective facility-community coordination of mother/baby follow-up would be challenged by a critical shortage of VHWs. The facility was not fully utilizing the stipend-supported (but otherwise voluntary) positions allocated through the MOH. They recruited and trained an additional 46 VHWs, bringing their catchment area total to 58. This included replacing several VHWs who were unable to deliver the full set of VHW responsibilities due to age and limited mobility. In all six facilities, MDTs identified that women testing HIV negative were not scheduled for HIV re-testing. Each MDT developed a monitoring strategy to ensure repeat testing per MOH guidelines. Table 2 summarizes an example range of health systems gaps identified and MDT-derived solutions employed to address them.

**Table 2:** Example Outcomes of MDT Gap Identification and Solution Implementation to Improve MCH outcomes

System Gap Identified	Solution Employed	Facility Type
Long wait times between MCH service points due to women taking their own specimens (blood and urine) to the laboratory and waiting to collect test results	MCH Nurses transport blood specimens and collect results. Urine tests conducted within MCH patient-provider interactions	Hospital
Pregnant women charged 1.00 Maloti to access toilet for pregnancy-confirmation urine specimen	Fees waived for pregnant women, cards identifying eligible patients developed and distributed	Hospital
Women testing HIV negative not scheduled for HIV re-testing	• Use of appointment system for HIV re-testing of HIV negative women • Monthly register review at MDT meetings to monitor repeat test scheduling • Use of MDT meetings to re-train, as necessary	All intervention facilities
Critical community-based staff shortage hinders patient tracking	Identification and training of 46 new VHWs, increasing capacity to 58 VHWs, total	Hospital
Minimal collaboration between facility and community-based PMTCT/MCH service providers	• Improved communication between facility and community-based staff members through MDT • Consistent use of MOH patient tracking tool during home visits • Creation of facility-specific referral tool for remote health center	Health Center

In an adaption on the single MOH tracking tool for improved facility and community-based MCH/PMTCT provider coordination, the MDT of a facility located in a particularly remote, mountainous area with poor telephone network reception and accessibility created an additional facility-specific referral form. The form was handed to every woman attending their first ANC visit. Women were instructed to give the referral form to their local VHW, who then used the interaction to build rapport and employ the key message job aids. The VHW brought the forms to the monthly VHW meeting where a LENASO linkage coordinator or facility ANC nurse documented successful referrals and identified missed referrals. Peer M2M attempted to telephone missed referrals. Updates were shared at monthly MDT meetings.

#### Lessons learned: enablers and challenges

In all six intervention health facilities, there was active involvement of key senior management decision makers, which facilitated ownership of IMPROVE intervention initiatives. Administrators and senior nurses actively participated in the initial meetings and encouraged the other staff. All key stakeholders involved in the facility and community follow-up of invited mothers and children agreed to participate in these meetings. Despite buy-in, logistical realities of coordination remained difficult. For example, variation in demands from donor agencies meant that stakeholder priorities, targets and schedules were inconsistent. This challenged MDT meeting scheduling and attendance. Additionally, transfers, retrenchment or staff turnover of key MDT members weakened some teams. However, in several facilities, implementation of quality improvement activities and monitoring led to periods when MDT meetings were more frequent than monthly, demonstrating that progress and feedback loops may encourage participation despite challenges.

Formal discussion led by the MDTs identified cadres had different systems for patient follow-up within a facility, and communication between cadres was poor. Efforts to promote broader coordination and engagement through the curriculum training, systems-thinking skills-based approaches and regular MDT meetings revealed the nature and extent of the challenge, creating a foundation for improved coordination. Routine assessment of community-based follow-ups through the MDT supported improved use of tracking tools and identification of sub-optimally performing community-based HCWs. This led to community-based HCW re-training or replacement. Communication between all entities with active implementation roles, including LENASO, M2M, and facility-based HCWs, was important for effective collaboration, reducing duplication of activities and improving working relationships.

While there was a perception among staff members that patient experiences and facility-community coordination improved with MDTs, patient loss-to-follow-up persisted. Reasons for unsuccessful in-person tracking efforts included mobility of patients outside of the clinic catchment area, arduous mountain terrain inhibiting patient access via vehicle or travel on foot, and ongoing HIV stigma. It was particularly difficult to track movement of patients outside of the facility catchment area, as facility record systems were not linked and resources for those living or seeking care outside the facility catchment were not available. Remote mountainous terrain and poor roads around facilities inhibited phone service and regular follow-up by vehicle. While some community follow-up was done on foot, it was difficult to sustain. Finally, other women did not want to be visited by VHWs, as they did not wish for HCWs who were neighbors to know their HIV status. Mothers often preferred home visits from M2M peer HCWs over VHWs but M2M struggled to gain access to the community due to distance and limited staffing. One site’s approach had VHWs and M2M peers make joint initial visits then conduct separate follow-ups as necessary.

## Discussion

The IMPROVE study used a participatory process throughout intervention development and implementation to optimize the appropriateness, acceptability, and feasibility of a health-systems-level intervention for improving MCH and HIV/PMTCT outcomes. Implementation of each intervention component was feasible and benefited from having facility-specific adaptation opportunities inherent to the intervention implementation strategies. Full clinical results of the study will be presented in a separate manuscript. However, preliminary study data analysis showed a trend toward improved ART adherence and undetectable viral load among women living with HIV in the intervention compared to the control arm [32].

The MDTs were the backbone of the intervention, supporting use of the PHDP key message job aids and early community-based follow-up and counseling of women subsequent to their initial ANC visit. The coordination and monitoring roles of the MDT were central to sustaining the other intervention components through identification of implementation gaps and ways to address them, including re-training or replacement of HCWs and other facility-specific solutions. While training interventions are more common in low-income settings, fewer studies target organizational strategies to influence change [33]. MDTs may serve as an example of a promising approach to facilitating organizational-level change.

Initiating the MDTs through the participatory training provided a foundation to integrate multiple cadres operating at different power dynamics. This included explicitly establishing shared values and purpose through building relationships and coordination among previously uncoordinated MCH/PMTCT service delivery providers. Multidisciplinary teams may offer flexible yet accountable management structures, with more horizontal power and communication exchange than in traditional hierarchically organized health facilities. This is consistent with other research showing cross-cadre training of diverse health workers was found to be important for improving HCW interactions and establishing the effective teamwork necessary for coherent health systems operations [34]. Facility-specific quality improvement approaches have shown potential for improving MCH outcomes in other settings [35]. The ongoing MDT meetings provided a venue for sustained quality improvement efforts and iterative, facility-specific problem solving. Additionally, reviewing progress at MDT meetings helped HCWs feel appreciated for their efforts and improved motivation for ongoing work. Finally, explicit active monitoring of MOH policies and guidelines supported facility-level implementation of new practices, such as adoption of the community tracking tool and SOPs around frequency of ANC services.

Active participation of health facility leadership in intervention activities created a supportive environment for intervention implementation and adaptation. Leadership support has been shown to be critical in other patient-centered care initiatives [36]. The study team hypothesizes the longstanding relationship between the health facilities, the MOH, and EGPAF, combined with the Intervention Study Coordinator’s previous experience in leading successful public health initiatives, support receptivity to the intervention. Similarly, involvement of leadership in the intervention development and adaptation [37] and a clear role in implementation [38] supported their engagement in intervention activities.

Consistent with extant research on health system-level interventions, despite facility-specific adaptations to improve feasibility, acceptability and appropriateness among health workers, structural health systems challenges to successful intervention implementation remained. Staff turnover [39, 40] and difficulty in coordination and full participation of relevant HCWs due to competing donor priorities [41] were prime among these. While some staff turnover came from routine transfer of HCWs between MOH and CHAL facilities, shifting or termination from M2M and LENASO due to changes in donor funding was the main source of MDT-related staff changes. While MDTs improved coordination of MOH/CHAL and community-based partner organization efforts, the donor-funded partners remained accountable to donor priorities and targets. Differences in MDT-identified and donor-identified priorities led resulted in conflicting time and resource allocation requirements. Supporting facility-led priority setting through coordinated approaches may aid improved patient- centeredness and care outcomes. Additionally, a lack of systems for cross-catchment area linkages for follow-up of mother-baby pairs in a mobile patient population jeopardized retention in care and accuracy of reporting. Estimates from South Africa show that approximately one quarter of pregnant women living with HIV fail to link to ART services postpartum [42]. Among those who link to HIV care postpartum, 21% attend two or more clinics, with younger and unemployed women more likely to seek MCH care at multiple health facilities [42]. Supra-facility interventions to link patient records [43] are likely necessary to support improved facility-based coordination in follow-up of mobile mother-infant pairs across recommended postpartum visits including growth monitoring and immunizations.

### Limitations

This manuscript was intended to share the formative and implementation phases of the IMPROVE intervention. Results are qualitative, as quantitative metrics of changes in HCW motivation and relationship dynamics, such as trust between cadres, were not collected. However, qualitative rigor was supported by consistent documentation throughout implementation, and analysis and results being led by study implementers and was shared with study stakeholders for feedback. Patient-level outcome metrics associated with the health system intervention are disseminated in a separate study report [14].

### Study follow-up

While a formal assessment of intervention sustainability and institutionalization beyond the study period was outside the scope of the study, the IMPROVE Study Intervention Coordinator obtained informal reports from facility leadership on ongoing implementation at four of the six intervention sites in October 2021. The key counseling messages were still being used during facility and/or home-based counseling of pregnant and breastfeeding women at three of the four sites. MDT meetings continued beyond July 2019 (study conclusion) at two sites. In one site, they were held through January 2020; and, in the other, the meetings were combined with facility – VHW meetings, and held through March 2020. National COVID-19-related restrictions on gatherings suspended the meetings in both sites, with routine gatherings yet to resume as of the writing of this manuscript. Community-based follow-up of ANC clients continued in all four facilities. Reported variations from study practices in ongoing implementation at some facilities included follow-up after the recommended 14-day period post-ANC visit and VHW referrals without coordination through LENASO or M2M. While VHWs recruited under IMPROVE were retained through the health system and contributed to follow-up efforts, reduced external funding for M2M in some areas meant mentor mothers were no longer available. COVID-19 restrictions also halted in-person community tracking efforts in all facilities; however, phone-based follow-up continues at some sites. The revised community tracking tool developed through the study was not formally instituted by the MOH, therefore it is no longer in use. Additionally, one facility reported that facility-specific improvement strategies instituted through the MDT during the study, such as accompanying patients from the front gate to the clinic service access area to ensure smooth movement through the clinic, have continued. IMPROVE intervention scale-up to other sites has not been attempted given the COVID-19 pandemic and accompanying mitigation measures restricting group gatherings.

## Conclusions

The IMPROVE intervention with inherent facility-specific adaptation opportunities through participatory implementation practices was generally appropriate, acceptable, feasible, and well-received across a range of facilities in Maseru District. Feasibility was challenged, however, by staff turnover and loss of community representatives as donor funding shifted away from community organizations. Key sources of variability in implementation across sites included staffing levels (with more severely under-staffed sites prioritizing staff increases), extant coordination (with greater effort spent on facility-community alignment where systems were most disjointed), and leadership buy-in. MDT success was greater and more sustained with leadership support and investment of HCW time. The MDTs offered a platform for relationship-building between profession and lay HCW cadres, facility-specific quality improvement practices to increase patient-centeredness, and, importantly, provided HCWs with recognition of their challenges and efforts. Increased coordination and monitoring objectives of the MDTs created a platform that supported other interventions, including ongoing use of the PHDP messages and early follow-up and counseling of mothers after first ANC visit. Stronger health systems coordination mechanisms may support improved patient-centered care practices in similar settings and for other health services.

## Data Availability

The data that support the findings of this study are available from the Elizabeth Glaser Pediatric AIDS Foundation, Lesotho but restrictions apply to the availability of these data, which were used for the current study, and so are not publicly available. Data are however available from the authors upon reasonable request and with permission of the Lesotho Ministry of Health.

## Abbreviations

ANC: Antenatal care
ART: Antiretroviral therapy
CHAL: Christian Health Association of Lesotho
COVID-19: Coronavirus disease of 2019
EID: Early infant HIV diagnosis
EGPAF: Elizabeth Glaser Pediatric AIDS Foundation
FP: Family planning
HCW: Healthcare worker
HIV: Human immunodeficiency virus
IMPROVE: Integrated Management Team to Improve Maternal-Child Outcomes study
IRB: Institutional Review Board
LENASO: Lesotho Network of AIDS Service Organizations
M2M: Mothers to mothers
MCH: Maternal and child health
MDT: Multidisciplinary care management team
MOH: Ministry of Health
PMTCT: Prevention of mother-to-child HIV transmission
PHDP: Positive health, dignity, and prevention
PNC: Post-natal care
Project SOAR: Supporting Operational AIDS Research
SOP: Standard operating procedure
TOT: Training of trainers
USAID: United States Agency for International Development
VHW: Village health worker
